# Assessing Patient Safety Culture in a Secondary Care Hospital in Saudi Arabia Using the Hospital Survey on Patient Safety Culture Tool: A Cross-Sectional Study

**DOI:** 10.7759/cureus.98682

**Published:** 2025-12-08

**Authors:** Mohammad Irfan, Najla Al Motairi, Muna Bhutta, Abidah Parveen, Mohamed El Attar

**Affiliations:** 1 Emergency Medicine, Mid Yorkshire Teaching NHS Trust, Wakefield, GBR; 2 Quality Management and Patient Safety, Al Iman General Hospital, Riyadh, SAU; 3 Obstetrics and Gynaecology, Al Iman General Hospital, Riyadh, SAU; 4 Department of Pharmaceutical Sciences, Abbottabad University Of Science and Technology, Havelian, PAK; 5 General Surgery, Al Iman General Hospital, Riyadh, SAU

**Keywords:** communication openness, hsopsc, patient safety culture survey, saudi arabia, staffing

## Abstract

Background: A positive patient safety culture (PSC) is integral to reducing preventable harm and improving healthcare outcomes. In many low- and middle-income countries, there is a lack of structured measurement of PSC, hindering the identification of systemic weaknesses. This study assessed PSC in a secondary care public hospital in Riyadh, Saudi Arabia, using the Hospital Survey on Patient Safety Culture (HSOPSC) tool, with the aim of identifying strengths, weaknesses, and predictors of a robust safety culture.

Local Problem: PSC remains under-assessed in Saudi secondary care hospitals, with inadequate systems for event reporting and weak staffing policies.

Methods: A cross-sectional study using the bilingual (Arabic/English) HSOPSC Version 1.0 was conducted in a 200-bed secondary hospital. All eligible staff were surveyed electronically. Statistical analysis included descriptive statistics, Cronbach’s alpha for internal consistency, Spearman correlation, and multiple linear regression to identify predictors of overall safety culture. Kruskal-Wallis tests compared scores across professional roles.

Intervention: Survey dissemination was supported by departmental champions and aimed to build a baseline for future safety initiatives.

Results: Of 1,084 eligible staff, 613 completed the survey (57% response). Teamwork within units (85%) and frequency of events reported (85%) scored highest; staffing (43%) and communication openness (57%) were weakest. Regression identified non-punitive response to error, event reporting frequency, and communication openness as the strongest predictors of overall PSC (p < 0.001). Nurses reported significantly lower scores than physicians (p < 0.001).

Conclusions: The study highlights strong internal collaboration but emphasises the need for improved staffing and transparency. These results form the basis for targeted quality improvement aligned with national healthcare goals.

## Introduction

Problem description

Patient safety remains one of the most critical global priorities in healthcare. The World Health Organisation (WHO) estimates that approximately one in ten hospitalised patients worldwide experiences some form of harm related to unsafe healthcare practices, and nearly half of these adverse events are considered preventable [1]. In low- and middle-income countries, unsafe medical care is estimated to contribute to approximately 2.6 million deaths annually, primarily due to systemic deficiencies, communication breakdowns, and the underreporting of incidents [2]. These findings highlight the substantial human, ethical, and economic burden associated with preventable harm in healthcare delivery systems.

In the Kingdom of Saudi Arabia (KSA), patient safety has been embedded as a central component of the Vision 2030 National Transformation Programme, which seeks to enhance healthcare quality, transparency, and accountability [3]. The Ministry of Health (MOH) has mandated the implementation of safety standards, accreditation systems, and structured event-reporting mechanisms across hospitals. Despite these reforms, incidents of preventable harm persist, and several studies suggest that many healthcare professionals continue to work within environments characterised by underreporting, punitive cultures, and workforce shortages [4]. These ongoing challenges underline the urgent need for a systematic evaluation of PSC as a cornerstone of sustainable improvement.

Available knowledge

PSC represents the collective values, attitudes, and behaviours that influence how safety is prioritised and managed within an organisation [5]. A positive safety culture is marked by openness, trust, teamwork, and a willingness to learn from errors [6]. Conversely, a weak culture that is defined by fear of blame, poor communication, and hierarchical barriers discourages reporting and obstructs learning [7].

To measure PSC, several validated tools have been developed globally. One of the most widely used is the hospital survey on patient safety culture (HSOPSC), introduced by the Agency for Healthcare Research and Quality (AHRQ) in 2004 [8]. The HSOPSC assesses 12 composite dimensions of safety culture, including teamwork within units, staffing, feedback about errors, and non-punitive response to errors. Its reliability and validity have been confirmed across numerous healthcare settings [9,10].

Studies conducted in the United States have provided extensive benchmark data for PSC measurement. Following the 1999 report To Err Is Human, which estimated that medical errors accounted for up to 98,000 deaths annually, U.S. healthcare institutions placed renewed emphasis on safety systems and culture [11]. The AHRQ surveys on patient safety culture (SOPS) hospital database (2022), comprising responses from over 180,000 healthcare workers, reported that teamwork within units achieved an average 82% positive response rate, while “non-punitive response to error” and “staffing” scored significantly lower at 47% and 50%, respectively [12]. Furthermore, 55% of U.S. respondents reported no adverse events in the preceding 12 months, suggesting persistent underreporting despite well-established systems [12]. These findings mirror the global trend of strong teamwork but weaknesses in openness and staffing, demonstrating that safety culture remains a shared challenge across nations.

International studies further reinforce these patterns. Singer and Vogus highlighted that interventions enhancing psychological safety and teamwork could significantly reduce medical errors [13]. Similarly, Flin et al. emphasised that measuring and improving safety climate in healthcare must move beyond procedural compliance to fostering shared responsibility [14]. Despite structural and technological advances, cultural barriers remain deeply rooted within healthcare organisations globally.

In the Middle East and North Africa (MENA) region, PSC research has expanded but still trails behind Western counterparts. Studies from Kuwait, Lebanon, and Oman revealed consistent themes of strong intra-unit teamwork but deficiencies in communication openness, staffing adequacy, and event reporting [15-17]. Alqattan et al. found that while Kuwaiti hospitals exhibited collaborative teamwork, fear of blame limited reporting [15]. El-Jardali et al. reported similar barriers in Lebanese hospitals, identifying insufficient leadership support for safety [16]. Al Mandhari et al. found comparable trends in Oman’s Ministry of Health hospitals [17]. In primary care, Klemenc-Ketis and Kersnik also found suboptimal safety culture among healthcare staff, with poor communication openness being a key concern [18].

In Saudi Arabia, despite the modernisation of healthcare infrastructure, the cultural and behavioural dimensions of safety remain underexplored. Al-Ahmadi’s research revealed that healthcare professionals in Riyadh perceived safety initiatives as fragmented and inconsistent, with limited leadership visibility and inadequate training [19]. More recent evidence by Alzahrani and Jones showed that while awareness of safety principles has improved, open communication and non-punitive reporting remain underdeveloped [4]. Thus, assessing PSC systematically across Saudi hospitals is essential to identify strengths, address weaknesses, and guide policy within the Vision 2030 framework.

Rationale

Despite global advancements in patient safety, many healthcare systems, particularly in the Gulf region, lack institutionalised mechanisms for measuring and monitoring PSC. Without robust baseline data, it becomes difficult to benchmark progress, identify cultural barriers, or design targeted improvement strategies [5]. The HSOPSC provides a validated, structured framework for assessing organisational safety culture. By examining 12 dimensions, it enables healthcare leaders to identify areas of strength and weakness and compare perceptions among professional groups such as physicians, nurses, and allied health professionals.

Culture cannot be legislated; it must be cultivated through measurement, engagement, and reinforcement [20]. In hierarchical systems, where communication between senior and junior staff is often constrained, PSC assessments can uncover subtle yet significant barriers to safety [21]. Moreover, culture measurement supports accreditation readiness, leadership development, and alignment with value-based reimbursement models that increasingly link safety outcomes to funding. Hospitals that fail to assess and address safety culture risk perpetuate preventable harm, staff burnout, and patient dissatisfaction [22,23].

Aim of the study

Given this context, the present study aimed to measure the current level of patient safety culture in a secondary-care hospital in Riyadh, Saudi Arabia, using the HSOPSC tool; identify composite dimensions of safety culture that were perceived as organisational strengths versus those needing improvement; compare perceptions of safety culture across professional groups, including nurses, physicians, and allied-health staff; and determine the key predictors of overall patient safety culture ratings to inform future quality-improvement initiatives and staff-training strategies.

## Materials and methods

Context

The study was conducted in a 200-bed public hospital located in Riyadh, Saudi Arabia. The facility provides multidisciplinary services, including general medicine, surgery, obstetrics, and emergency care. No previous PSC assessments had been conducted at this site. Hospital leadership endorsed the study as part of an internal quality improvement initiative under the Vision 2030 Healthcare Transformation Framework [3].

Intervention

The intervention involved administering the HSOPSC version 1.0 to all hospital departments to establish a baseline measurement of safety culture. Departmental “safety champions” were appointed to facilitate engagement, encourage participation, and coordinate survey distribution. A bilingual (Arabic/English) version of the survey was selected, based on prior validation studies conducted in similar cultural and linguistic contexts [13].

Focus of the intervention

The evaluation examined patient safety culture across the 12 HSOPSC dimensions, comparing perceptions among professional groups, and identifying the strongest predictors of overall safety perception. The findings were used to design context-specific improvement strategies and to develop a continuous monitoring framework.

Measures

The HSOPSC v1.0 is an open-access instrument developed by the AHRQ [9]. The questionnaire is in the public domain; therefore, no permission was required. The composites of the survey include teamwork within units, communication openness, feedback about error, staffing, and non-punitive response to error, among others. Each item is scored on a 5-point Likert scale, ranging from “Strongly disagree” to “Strongly agree.” Negatively worded items were reverse-coded to ensure consistency. In addition to these composites, the survey includes two outcome variables: the overall safety grade assigned by respondents and the frequency of event reporting.

Data collection and analysis

Data were collected between March - August 2020. All eligible staff members (N=1084) received an electronic survey link via institutional email and the hospital’s internal WhatsApp communication group. Participation was voluntary and anonymous to encourage honest feedback.

Descriptive statistics were computed for demographic characteristics and for each of the 12 PSC dimensions. Cronbach’s alpha was used to test internal consistency and reliability, following methods outlined by Tavakol and Dennick [15]. Correlations between composite dimensions and the overall perception of safety were examined using Spearman’s rank correlation coefficient, consistent with non-parametric data assumptions [16]. A multiple linear regression analysis was conducted to identify independent predictors of PSC scores, while the Kruskal-Wallis H test was employed to compare PSC perceptions among physicians, nurses, and allied health professionals. All analyses were performed using SPSS version 22, following recommended analytical procedures for HSOPSC datasets [13].

Ethical considerations

Ethical approval was obtained from the hospital’s patient safety and risk management department and patient advocacy committee. participation was voluntary, and completion of the online survey was considered informed consent. All responses were collected anonymously, and no identifiable data were stored or reported.

## Results

Response rate and demographics

A total of 613 completed surveys were received from 1084 eligible staff, producing a 57% response rate. Nearly half of respondents were physicians (47%, n = 287), followed by nurses (36%, n=221) and allied health professionals (17%, n=105). The majority (83%, n=508) reported direct involvement in patient care, ensuring representation from those most engaged with frontline safety practices. This composition provided a robust cross-sectional profile of the hospital workforce for assessing patient safety culture (PSC) (Table 1).

Patient safety grade perceptions

When asked to rate their unit’s overall level of patient safety, 42% (n=259) of respondents described it as excellent and 37% (n=227) as very good. Meanwhile, 18% (n=112) considered it acceptable, and only 2% (n=13) rated it as poor or failing. Collectively, approximately 79% (n=486) expressed a positive perception of their unit’s safety climate, suggesting widespread confidence in safety systems, although one in five participants highlighted ongoing areas for improvement (Table 1).

**Table 1:** Patient safety grading, incident reporting and respondent characteristics Descriptive statistics summarising the demographic profile and safety perceptions of participants (N = 613). The response rate was 57% of the total eligible staff (N = 1084). Percentages represent the proportion of positive responses within each category. All data were analysed descriptively using SPSS v22; no inferential statistics were applied for this summary table.

Respondent Characteristics			Response Rate (Total Population=1084)	Respondent Characteristics			N=613
Physicians	47%	287	57% (N=613)	Are you in direct contact with the patient?			
Nurses	36%	221		Yes		83%	508
Others (Pharmacy, Lab, Radiology, Dietician, Admin staff, etc)	17%	105		No		17%	105
Patient Safety Grading		N=613	Patient Safety Culture-Percent Positive	Incident Reporting		N=613	Patient Safety Culture-Percent Positive
Excellent	42%	259	79% (n=486)	20 or More Events Reported	5%	28	56% (n=346)
Very Good	37%	227		11 -20 Events Reported	6%	38	
Acceptable	18%	112		6-10 Events Reported	14%	89	
Poor	2%	13		3-5 Events Reported	13%	77	
Failing	0.33%	2		1-2 Events Reported	19%	114	
				No Event Reported	43%	267	

Incident reporting frequency

Despite the high overall perception of safety, patterns in self-reported event reporting indicated possible underreporting. Forty-three percent of respondents stated they had not reported any safety events in the preceding 12 months, while 19% reported one or two events. In contrast, only 5% of participants had reported twenty or more events. These findings suggest a persistent reporting gap within the institution, possibly linked to uncertainty about reporting systems or apprehension regarding blame, warranting targeted cultural interventions (Table 1).

Composite patient safety culture scores

**Figure 1:**
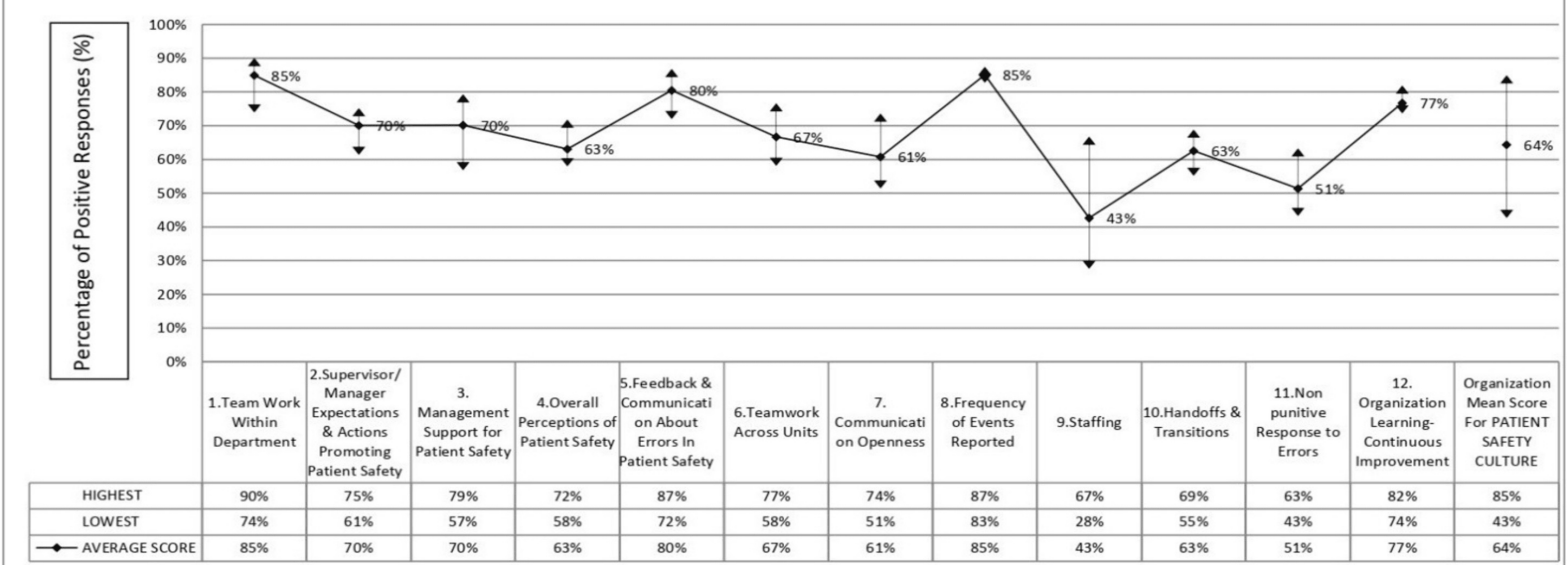
Patient safety culture survey: Safety composites and organisational safety culture- mean percent positive score Mean percent-positive responses for each of the twelve HSOPSC v1.0 dimensions. The Y-axis represents the percentage of positive responses (%). Data are displayed as average composite scores with the highest and lowest values indicated for each domain. Statistical analysis: Descriptive analysis was performed to determine the mean and range of positive responses for each safety composite. No inferential statistical test was applied for Figure 1, as the graph represents descriptive summary data only. HSOPSC: Hospital Survey on Patient Safety Culture; AHRQ: Agency for Healthcare Research and Quality

Analysis of the twelve HSOPSC composites revealed notable variability across safety domains (Figure 1, Table 2). The highest mean positive responses were observed for teamwork within units (85%), and frequency of events reported (85%), followed by feedback and communication about error (80%) and organisational learning and continuous improvement (77%). In contrast, three composites scored below the 60% threshold generally considered acceptable for a strong safety culture: Communication openness (57%), non-punitive response to error (59%), and staffing (43%). These results indicate that while interpersonal collaboration and feedback mechanisms are relatively strong, systemic barriers such as inadequate staffing and fear of blame remain persistent challenges (Table 2).

**Table 2:** Patient safety culture questionnaire Responses to the Hospital Survey on Patient Safety Culture (HSOPSC v1.0) were developed by the Agency for Healthcare Research and Quality (AHRQ, USA)—an open-access, public-domain instrument. The table presents mean, median, and standard deviation values for each of the 12 composite dimensions (Cronbach’s α = 0.916 for 42 items). Percent-positive scores were calculated as the proportion of “Agree/Strongly Agree” (or “Always/Most of the time” for frequency questions) responses. Negatively worded items were reverse-coded (R). Internal-consistency reliability for each composite is shown by Cronbach’s α values in parentheses. Statistical analysis included descriptive statistics and reliability testing (Cronbach’s α); normality was confirmed using the Shapiro–Wilk test. Standard Error of Skewness for each composite: 0.099, Standard Error of Kurtosis for each composite: 0.197. Abbreviations: SA: Strongly Agree, A: Agree, N: Neutral, D: Disagree, SD: Strongly Disagree, AR: Always Reported, MFR: More Frequently reported, LFR: Less Frequently Reported, NR: Never Reported, (R): Reverse Coded Questions

Patient Safety Culture- 12 Safety Composites - 42 Questions (Cronbach’s Alpha-0.916 – 42 Items)	SA (5)	A (4)	N (3)	D (2)	SD (1)	Percent Positive Culture	Mean	Median	Std. Dev
1. Team Work Within Department (Cronbach’s Alpha- 0.691)							4.115	4.25	0.638
Q1.1. Do people support one another in this unit?	147	397	35	16	18	89% (544)			
Q1.2. When a lot of work needs to be done quickly, we work together as a team to get the work done.	107	426	38	21	21	87(533)			
Q1.3. In this unit, people treat each other with respect?	481	71	33	18	10	90% (552)			
Q1.4. When one area in this unit gets really busy, others help out?	165	288	100	26	34	74% (453)			
2. Supervisor/Manager Expectations & Actions Promoting Patient Safety (Cronbach’s Alpha- 0.452)							3.685	4	0.665
Q2.1. My Supervisor / Manager says a good word when he/she sees a job done according to established patient safety procedures?	44	417	62	73	17	75% (461)			
Q2.2. My Supervisor/ Manager seriously considers staff suggestions for improving patient safety?	66	410	65	55	17	78% (476)			
Q2.3. Whenever pressures build up, my Supervisor/ Manager wants us to work faster, even if it means taking shortcuts? (R)	33	130	74	107	269	61% (163)			
Q2.4. My Supervisor / Manager overlooks/fails to notice patient safety problems that happen over and over? (R)	43	72	92	274	132	66% (406)			
3. Management Support for Patient Safety (Cronbach’s Alpha- 0.640)							3.701	4	0.829
Q3.1. Hospital management provides a work climate that promotes patient safety?	148	308	73	43	41	74% (456)			
Q3.2. The actions of hospital management show that patient safety is a TOP PRIORITY?	99	387	56	64	7	79% (486)			
Q3.3. Hospital management seems interested in patient safety ONLY AFTER an adverse event happens? (R)	18	184	63	172	176	57% (348)			
4. Overall Perception of safety (Cronbach’s Alpha- 0.339)							3.617	3.75	0.674
Q4.1. It is just by chance that more serious mistakes don't happen around here? (R )	61	112	88	195	157	57% (352)			
Q4.2. Is patient safety ever sacrificed to get more work done?	318	122	68	85	20	72% (440)			
Q4.3. Our procedures and systems are good at preventing errors from happening?	17	383	85	119	9	65% (400)			
Q4.4. We have patient safety problems in this unit? (R )	21	171	66	209	146	58% (355)			
5. Feedback & Communication About Errors In Patient Safety (Cronbach’s Alpha- 0.655)							4.079	4.33	0.816
Q5.1. We are given feedback about changes put into place based on event reports?	143	298	76	71	25	72% (441)			
Q5.2. We are informed about errors that happen in this unit?	350	157	54	23	29	83% (507)			
Q5.3. In this unit, we discuss ways to prevent errors from happening again?	294	238	40	2	39	87% (532)			
6. Teamwork Across Units (Cronbach’s Alpha-0.644)							3.653	3.75	0.8
Q6.1. Hospital units DO NOT cooperate well with each other? (R)	19	141	97	75	281	58% (356)			
Q6.2. Is there good cooperation among hospital units that need to work together?	96	315	86	72	44	67% (411)			
Q6.3. Is it OFTEN UNPLEASANT to work with staff from other hospital units? (R)	33	110	72	244	154	65% (398)			
Q6.4. Hospital units WORK WELL TOGETHER to provide the BEST CARE for patients?	193	277	72	53	18	77% (470)			
7. Communication Openness (Cronbach’s Alpha- 0.609)							3.502	3.667	0.967
Q7.1. Staff will freely speak up if they see something that may negatively affect patient care?	146	305	57	82	23	74% (451)			
Q7.2. Staff feel free to question the decisions or actions of those with more authority?	58	257	115	70	113	51% (315)			
Q7.3. Are staff afraid to ask questions when something does not seem right? (R)	64	130	68	67	284	57% (351)			
8. Frequency of Events Reported (Cronbach’s Alpha-0.869)	AR (4)	MFR (3)	LFR (2)	NR (1)			2.879	3.000	1.038
Q8.1. When a mistake is made, but is caught and corrected before affecting the patient. How often is this reported?	285	78	158	92		85% (521)			
Q8.2. When a mistake is made, but has no potential to harm the patient. How often is this reported?	258	68	181	106		83% (507)			
Q8.3. When a mistake is made that could harm the patient, but does not, how often is this reported?	284	92	160	77		87% (536)			
9. Staffing (Cronbach’s Alpha-0.471)							2.931	3	0.838
Q9.1. Do we have enough staff to handle the workload?	40	147	27	67	332	31% (187)			
Q9.2. Staff in this unit work longer hours, which is best for patient care? (R)	114	158	60	223	58	46% (281)			
Q9.3. We use more temporary staff THAT MAY NOT BE best for patient care? (R)	40	117	47	16	393	67% (409)			
Q9.4. We work in "Crisis Mode" trying to do too much, too quickly? (R)	72	300	72	118	51	28% (169)			
10.Handoffs & Transitions (Cronbach’s Alpha-0.703)							3.617	3.75	0.855
Q10.1. Things "fall between the cracks" when transferring patients from one unit to another? (R)	33	107	97	91	285	61% (376)			
Q10.2. Important patient care information is OFTEN LOST during shift changes? (R)	10	92	89	339	83	69% (422)			
Q10.3. Problems OFTEN OCCUR in the exchange of information across hospital units? (R )	25	171	79	238	100	55% (338)			
Q10.4. Are shift changes problematic for patients in this hospital? (R)	42	84	88	215	184	65% (399)			
11. Non-punitive Response to Errors (Cronbach’s Alpha-0.632)							3.015	3	1.191
Q11.1. Staff feel like their mistakes are held against them? (R)	77	159	85	209	83	48% (292)			
Q11.2. When an event is reported, it feels like the person is being written up, not the problem? (R)	208	66	74	116	149	43% (265)			
Q11.3. Incident report is never considered in annual evaluation of the staff in their personal files.	26	362	87	119	19	63% (388)			
12. Organization Learning-Continuous Improvement (Cronbach’s Alpha-0.824)							4.06	4	0.758
Q12.1. We are actively doing things to improve patient safety?	267	235	90	13	8	82% (502)			
Q12.2. Mistakes have led to positive changes here?	190	268	113	34	8	75% (458)			
Q12.3. After we make changes to improve patient safety, we evaluate their effectiveness?	193	258	135	22	5	74% (451)			

Professional Differences in Safety Culture Perceptions

**Figure 2:**
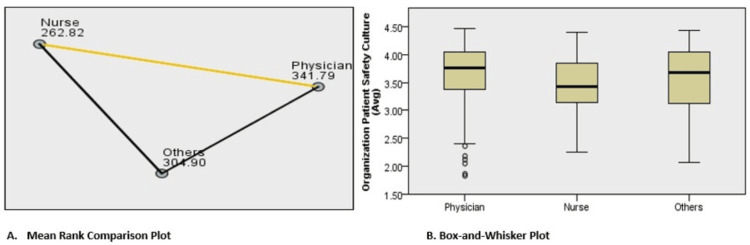
Comparison of patient safety culture scores across professional groups. A. Mean Rank Comparison Plot showing the relative mean ranks of PSC scores among physicians, nurses, and allied-health staff, as derived from the Kruskal–Wallis H test. Higher mean ranks correspond to more positive perceptions of patient safety culture. Physicians had the highest mean rank (341.79), followed by Others (allied-health staff - 304.90), while nurses reported the lowest (262.82). (B) Box-and-Whisker Plot illustrating the distribution of PSC scores across professional groups. The central horizontal line represents the median, the box indicates the interquartile range (IQR), and whiskers depict the variability outside the upper and lower quartiles. Outliers are shown as individual points. Statistical analysis using the Kruskal–Wallis H test indicated significant differences among groups (p < 0.001). Detailed test statistics and post-hoc pairwise comparisons are provided in Table 3. Analyses were conducted using SPSS v22.0, with significance defined as p < 0.05. Abbreviations: Avg: Average; IQR: Interquartile range; PSC: Patient safety culture

A Kruskal-Wallis H test demonstrated a statistically significant difference in overall PSC scores among professional groups (H = 24.844, p < 0.001). Post-hoc pairwise comparisons revealed that nurses scored significantly lower than physicians (p < 0.001), a pattern often attributed to differences in workload and decision-making autonomy. Differences between nurses and allied health professionals were marginal (p = 0.045; adjusted p = 0.135*), while no significant difference was observed between allied health staff and physicians (adjusted p = 0.203) (Table 3, Figure 2).

**Table 3:** Kruskall-Wallis test Kruskal–Wallis H test assessing differences in overall patient safety culture scores among professional groups (Physicians, Nurses, and Other, which are allied-health staff). The analysis demonstrated a statistically significant difference between groups (H = 24.844, df = 2, p < 0.001). Post-hoc pairwise comparisons using Dunn’s test with Bonferroni correction indicated that nurses reported significantly lower PSC scores than physicians (p < 0.001). Differences between nurses and other staff were borderline significant (p = 0.045; adjusted p = 0.135), whereas no significant difference was observed between allied-health staff and physicians (p=0.068, adjusted p = 0.203). All analyses were conducted using SPSS version 22.0. A two-tailed p-value < 0.05 was considered statistically significant. Abbreviations: PSC: Patient Safety Culture; df: Degrees of Freedom; Sig: Significance; Std: Standard; Asymp Sig: Asymptotic significance; Adjusted Sig: Bonferroni-adjusted significance value.

			Total (N)	Test Statistic	df	Asymptomatic Sig.(2-sided Test)
			613	24.844	2	0.000
POST HOC TEST FOR PAIRWISE COMPARISON	SAMPLE 1-SAMPLE 2	Test Statistic	Std Error	Std. Test Statistic	Sig.	Adjusted Sig.
	Nurse – Physician	78.970	15.849	4.983	0.000	0.000
	Nurse- Others	42.081-	20.991	2.005-	0.045	0.135
	Others- Physician	36.889	20.199	1.826	0.068	0.203

Predictive Analysis of PSC Determinants

Multiple linear regression analysis identified three independent predictors that collectively explained 79% of the variance in overall PSC scores (R² = 0.79, p < 0.001*). The predictors were the frequency of events reported, the non-punitive response to error, and communication openness. These factors emerged as the strongest contributors to a positive safety culture, underscoring that transparent reporting, open dialogue, and an environment free of blame are key determinants of sustainable patient-safety improvement [5,24,25].

## Discussion

Summary of key patterns

The study revealed commendable teamwork and feedback mechanisms but persistent weaknesses in staffing, open communication, and fear of blame. Although 79% of respondents rated unit safety as high, low incident-reporting rates suggested underreporting. Nurses consistently reported lower PSC perceptions than physicians, underscoring hierarchical and workload-related barriers [6-8,11,12].

Interpretation of the findings

The overall pattern aligns with findings from neighbouring Gulf states, where teamwork within units typically scores highest and non-punitive response and staffing score lowest [6-8]. In hierarchical healthcare environments, strong intra-team cohesion can sometimes act as a coping mechanism for limited organisational trust rather than a sign of systemic safety.

Underreporting of incidents remains a universal challenge. Studies from Canada and Jordan demonstrated that fear of blame and limited feedback mechanisms were key deterrents to event reporting [18,19]. The same paradox was observed here: favourable safety ratings coexisted with limited reporting behaviour, implying that positive self-assessment may reflect perceived competence rather than genuine openness to error disclosure.

Staffing shortages, identified as the lowest-scoring domain (43%), are a well-documented determinant of adverse outcomes and staff burnout [20,21]. International evidence from Griffiths et al. and Aiken et al. confirms that insufficient nurse-to-patient ratios correlate directly with increased mortality, infection rates, and reduced job satisfaction [21,22]. Chronic understaffing also undermines team resilience, inhibits incident learning, and accelerates turnover.

The low score for non-punitive response to error (59%) echoes global concerns that punitive cultures suppress open reporting [5,6,24]. As Manser noted, teamwork and trust are essential for effective error management but quickly erode in environments where staff fear blame [24]. Leadership walk-rounds, anonymous reporting systems, and visible corrective feedback can help rebuild psychological safety.

Communication openness, another weak area (57%), is fundamental to preventing harm. Hierarchical settings often restrict upward communication, particularly among junior clinicians and nurses [19,26]. Although feedback mechanisms scored high (80%), the ability to voice immediate concerns appears constrained-a pattern also observed in studies from Oman, Lebanon, and primary care settings in Europe [7,8,27].

These findings are consistent with Western trends and recent U.S. data. The 2022 AHRQ SOPS report similarly identified strong teamwork (82%) but weak non-punitive culture (47%) [16]. Even in well-resourced systems, reluctance to report errors persists, underscoring that transformation in safety culture depends more on leadership and trust than technology. “High Reliability Organisation” (HRO) frameworks promote learning from failure and resilience rather than punishment: principles highly applicable to Middle Eastern healthcare systems. Similarly, the “Just Culture” model encourages accountability without blame, supporting balanced responses to error that sustain staff confidence [18].

The parallel between U.S. and Saudi findings is instructive: both demonstrate strong teamwork but limited openness. Improving PSC, therefore, requires not only procedural enhancement but also psychological safety and system-level redesign.

Differences by professional role

The gap between physicians and nurses in perceived safety culture deserves particular attention. Studies by Al-Ahmadi and Alzahrani & Jones demonstrated that Saudi nurses often face heavier workloads, limited autonomy, and weaker managerial support than physicians [11,12]. Similar disparities have been reported internationally, where nurses’ proximity to patient care exposes them to greater safety risks but affords less authority to address them [20,23].

Ahmed et al. and Alzahrani & Jones further observed that frontline professionals, especially nurses, are often underrepresented in safety committees and improvement initiatives [12,28]. When staff perceive a disconnect between leadership priorities and operational realities, engagement diminishes and speaking-up behaviour declines. Addressing these gaps requires tailored interventions that target communication, workload, and empowerment across professional groups.

Strengths and innovation

This study represents one of the few comprehensive assessments of PSC in Saudi secondary care using a validated, bilingual HSOPSC instrument. Inclusion of multiple professional categories enabled a holistic understanding of organisational culture. The analytical framework combining descriptive, non-parametric, and regression analyses provided an in-depth exploration of the determinants of safety culture.

Use of hospital communication platforms (email and WhatsApp) facilitated accessibility and participation, consistent with best practices in contemporary survey methodology. The resulting dataset provides a valuable baseline for iterative quality improvement cycles. By identifying low-performing dimensions such as staffing and openness, hospital leaders can design targeted interventions and monitor progress through periodic reassessment.

Limitations

This study has several limitations. First, it is cross-sectional in nature, capturing perceptions at a single point in time. Longitudinal follow-up will be required to assess whether interventions based on these findings lead to sustained improvements. Second, while the response rate was acceptable, self-selection bias is possible; those who chose to respond may have had stronger opinions (positive or negative) than those who did not.

Third, the survey relied entirely on self-report measures, which are subject to social desirability bias. Despite the anonymity of responses, staff may still have rated their environments more positively or avoided extreme negative responses due to perceived repercussions. Fourth, the study was conducted in a single secondary care hospital, and the findings may not be generalized to other hospital types, such as tertiary or primary care facilities, or to private institutions.

The findings underline the need for a comprehensive, institution-wide strategy to strengthen patient safety culture (PSC). Three interrelated priorities emerge from this study. First, it is essential to foster a non-punitive culture in which staff are encouraged and rewarded for reporting errors, while clearly distinguishing between human error, risky behaviour, and negligence. This approach aligns with the principles described by Vogus and Sutcliffe, who demonstrated that trusted leadership and learning-oriented systems are associated with a reduction in adverse events [25]. Second, communication openness must be enhanced by equipping leaders to invite feedback, conduct structured debriefings, and flatten hierarchies that often inhibit real-time disclosure of safety concerns. Similar initiatives implemented in Canadian and Omani hospitals have been shown to improve both staff morale and reporting rates [18,8]. Third, optimising staffing and resource allocation is vital to ensuring safe care. Adequate staffing levels should be aligned with patient acuity and workload, and frontline clinicians should be actively involved in workforce planning to ensure feasibility and engagement [20-23].

Leadership visibility, transparency, and timely feedback loops should also be integrated into the hospital’s governance framework to sustain improvement. As Pronovost et al. cautioned, measurement without visible action breeds cynicism; however, when performance metrics inform accountability and learning, they become powerful instruments for cultural and operational change [29].

Next steps

The findings of this study have been shared with hospital leadership and have guided the development of a targeted quality improvement (QI) roadmap. This roadmap incorporates several ongoing initiatives, including department-specific feedback sessions, regular safety huddles, the integration of PSC metrics into organisational performance dashboards, leadership safety walkarounds, and the annual re-administration of the HSOPSC tool to monitor progress over time. Collectively, these actions aim to embed continuous learning and transparent feedback into the hospital’s governance processes. They are closely aligned with the World Health Organisation’s Framework for Integrated, People-Centred Health Services, which recognises patient safety as a core element of healthcare quality and emphasises continuous assessment, staff engagement, and system-wide learning [30].

## Conclusions

This study provides a comprehensive evaluation of PSC in a secondary care hospital in Saudi Arabia, revealing both notable strengths and areas requiring urgent improvement. High ratings in teamwork, organisational learning, and feedback about errors demonstrate a solid foundation of collaboration and a willingness to improve. However, persistently low scores in staffing adequacy, communication openness, and non-punitive response to error indicate underlying systemic challenges that may threaten the sustainability of patient safety initiatives.

The identification of key predictive dimensions, particularly the frequency of event reporting, openness in communication, and freedom from blame, offers actionable insights for targeted quality improvement efforts. The observed differences in perceptions across professional groups, especially between nurses and physicians, highlight the need for role-specific strategies that address workload, communication, and empowerment. By employing a validated, open-access tool and engaging a diverse range of healthcare staff, this study establishes a robust baseline for future interventions and contributes valuable insight into professional differences in patient safety perceptions within the Saudi healthcare context.
